# Interactions Between Specific Immune Status of Pregnant Women and SARS-CoV-2 Infection

**DOI:** 10.3389/fcimb.2021.721309

**Published:** 2021-08-12

**Authors:** Ruirong Chen, Shaofen Zhang, Sheng Su, Haiyan Ye, Haihua Shu

**Affiliations:** ^1^Department of Anesthesiology, Guangdong Provincial People’s Hospital, Guangdong Academy of Medical Sciences, Guangzhou, China; ^2^The Second School of Clinical Medicine, Southern Medical University, Guangzhou, China; ^3^Department of Gynaecology and Obstetrics, Guangdong Provincial People’s Hospital, Guangdong Academy of Medical Sciences, Guangzhou, China; ^4^Department of Cardiology, Guangdong Cardiovascular Institute, Guangzhou, China

**Keywords:** SARS-CoV-2, COVID-19, pregnant women, immune system, treatment

## Abstract

Severe acute respiratory syndrome coronavirus 2 (SARS-CoV-2) is the pathogen responsible for the Coronavirus Disease 2019 (COVID-19) global pandemic. Because it is a new and highly contagious coronavirus, most people, especially pregnant women, lack immunity. It is therefore important to understand the interaction between why pregnant women are susceptible to SARS-CoV-2 and the specific immune systems of pregnant women. Here, we provide an overview of the changes that occur in the immune system during pregnancy, the activation and response of the immune system in pregnant women with COVID-19, adverse pregnancy outcomes in pregnant women with COVID-19, and the treatment and prevention of COVID-19 in this population.

## Introduction

Coronavirus Disease 2019 (COVID-19) is an acute respiratory infectious disease caused by severe acute respiratory syndrome coronavirus 2 (SARS-CoV-2), which has become a public health emergency of international concern in recent times. According to past epidemiological data, pregnant women are significantly more susceptible to influenza viruses and have higher morbidity from these infections than non-pregnant women (Zhao et al., 2020). With further expansion of the pandemic, pregnant women have gradually been found to be susceptible to SARS-CoV-2, which is mainly related to the special immune states of women during pregnancy (Zhu et al., 2020). Although pregnant women are at an increased risk for SARS-CoV-2 infection, the majority of pregnant women with COVID-19 have mild symptoms; about one-fifth of them develop moderate or severe disease (Andrikopoulou et al., 2020; Islam et al., 2020). The results of a meta-analysis of pregnant women and COVID-19 (n=236) showed that 51% of pregnant women infected with COVID-19 had fever (*vs.* 91% non-pregnant patients) and 31% had cough (*vs.* 67% non-pregnant patients) (Gao et al., 2020). On the other hand, studies have shown COVID-19 combined with pregnancy can lead to premature delivery, fetal distress, fetal vascular perfusion, premature rupture of membranes, and other adverse pregnancy events (Chen H. et al., 2020). Although the treatments for COVID-19 are evolving, health care providers do not have specific treatments for pregnant women. Understanding the changes that occur in the immune system during pregnancy and the interaction between the immune system and COVID-19 is extremely important, because each key link is expected to become a potential target of COVID-19, and provides new methods and ideas for the treatment of COVID-19 in pregnant women.

## Major Changes in the Immune System in Pregnant Women

Compared to non-pregnant women, pregnant women have a unique state of immunity. This status can affect how pregnant women respond to viral infections (Silasi et al., 2015), and SARS-CoV-2 is no exception. The main changes are summarized below (**Table 1**):

**Table 1:** “Physiological changes in pregnant women” and “Effects of SARS-CoV-2”.

Cell/Receptor/Cytokine	Function	Physiological changes in pregnant women	Effects of SARS-CoV-2
CD3+ T cell	Constructs TCR/CD3 complex and stabilizes its structure Participates T-cell activation signal transduction (Mesner et al., 2020)	Decreases in level and activity when stimulated	Weakens cellular immunity, and SARS-CoV-2 is more likely to invade
CD4+ T cell	Participates in the TCR antigen recognition process of TH cells (Horkova et al., 2020)	Decreases in level and activity when stimulated Differentiates to Th2 phenotype -The activity of Th 1 phenotype decreases	Weakens cellular immunity, and SARS-CoV-2 is more likely to invade Weakens humoral immunity, and the secretion of antibodies is reduced
CD8+ T cell	Killing effect on some antigens such as viruses and tumor cells (Horkova et al., 2020)	Decreases in level and activity when stimulated	Weakens the ability of CD8+ T cells to kill target cells, and SARS-CoV-2 is more likely to invade
NK cell	Identifies target cells, killing agents (Huntington et al., 2020)	Decreases	Weakens innate immune response to SARS-CoV-2
DC cell	Initiates, regulates, and maintains the immune response Activates NK cell (Chen et al., 2016)	mDC/pDC↑ pDC↓	Weakens the initiation of adaptive immune response to SARS-CoV-2 Reduced activation of NK cell weakens the innate immune response to SARS-CoV-2
Neutrophil	Chemotaxis Phagocytosis -Bactericidal action (Grunwell et al., 2019; Jaillon et al., 2020)	Reduces phagocytosis	SARS-CoV-2 is more likely to invade
Monocytes	Phagocytosis Antigen presentation (Guilliams et al., 2018)	Reduces phagocytosis	SARS-CoV-2 is more likely to invade
IFN-γ	Inhibitory angiogenesis Increases cytotoxic effects Promotes the differentiation of CD4+T cells to Th1 type and maintains the stability of Th1 phenotype Chemotaxis and activation of neutrophils, monocytes, and macrophages (Haep et al., 2015; Alspach et al., 2019)	Decreases	Reduces inflammatory response and reduces symptoms after SARS-CoV-2 infection Delays virus clearance and increases infection rate
VEGF	Promotes the proliferation of vascular endothelial cells Induction of angiogenesis Increased vascular permeability (Holmes et al., 2007)	Decreases	Reduces inflammatory response and reduces symptoms after SARS-CoV-2 infection Delays virus clearance and increases infection rate
IL-1α	Activates CD4+T cells, B cells, NK cells, neutrophils, monocytes Pyrogen 1 (Mantovani et al., 2019)	Decreases	Reduces inflammatory response and reduces symptoms after SARS-CoV-2 infection Delays virus clearance and increases infection rate
IL-1β	Activates CD4+T cells, B cells, NK cells, neutrophils, monocytes Pyrogen 1 (Mantovani et al., 2019)	Decreases	Reduces inflammatory response and reduces symptoms after SARS-CoV-2 infection Delays virus clearance and increases infection rate
IL-2	Stimulates and activates the proliferation of B and T cells (Watson et al., 1979; Wilson and Livingstone, 2008) Inhibits differentiation of the Th17 cells (Laurence et al., 2007)	Decreases	Reduces inflammatory response and reduces symptoms after SARS-CoV-2 infection Delays virus clearance and increases infection rate
IL-4	Stimulates and activates the proliferation of B and T cells Stimulates CD4+ T cells to differentiate into the Th2 phenotype Against the action of INF-γ-activated macrophages (Junttila, 2018; Franke et al., 2020)	Increases	Reduces inflammatory response and reduces symptoms after SARS-CoV-2 infection
IL-6	Stimulates the synthesis of APP in liver cells Stimulates the proliferation of activated B cells and secretes antibodies Stimulates T-cell proliferation and CTL activation (Hirano, 2021)	Decreases	Reduces inflammatory response and reduces symptoms after SARS-CoV-2 infection Delays virus clearance and increases infection rate
IL-10	Inhibition of macrophage response Suppresses cellular immunity Promotes humoral immunity (Walter, 2014)	Increases	Reduces inflammatory response and reduces symptoms after SARS-CoV-2 infection Delays virus clearance and increases infection rate
IL-12	Stimulates the proliferation of activated T cells, Promotes the differentiation of T0 cells to T1 cells Induces cytotoxic activity of CTL and NK cells 6 (Trinchieri, 2003; Furue et al., 2019)	Decreases	Delays virus clearance and increases infection rate
IL-13	Inhibits the release of IL-1, IL6, and other pro-inflammatory cytokines from monocytes Promotes the immune response of TH cells (Hart et al., 1999)	Increases	Reduces inflammatory response and reduces symptoms after SARS-CoV-2 infection Delays virus clearance and increases infection rate
IL-17	Mediates tissue inflammation (Amatya et al., 2017)	Decreases	Reduces inflammatory response and reduces symptoms after SARS-CoV-2 infection Delays virus clearance and increases infection rate
TNF	Enhances cytotoxicity Enhances phagocytosis of neutrophils Damages endothelial cells (Virdis et al., 2011; Brenner et al., 2015)	Decreases	Reduces inflammatory response and reduces symptoms after SARS-CoV-2 infection Delays virus clearance and increases infection rate
TGF-β	Inhibits proliferation of immunocompetent cells Inhibits lymphocyte differentiation Inhibits cytokine production (Travis and Sheppard, 2014)	Increases	Reduces inflammatory response and reduces symptoms after SARS-CoV-2 infection Delays virus clearance and increases infection rate
ACE2	Promotes angiotensin conversion Key receptors on cell surface that bind to SARS-CoV-2 (Levy et al., 2008; Jamilloux et al., 2020; Zhou et al., 2020)	Expression and activity are increased	Chance of SARS-CoV-2 invasion increases Enhances the protective effect of ACE2 on the lung after SARS-CoV-2 infection
Ang 1-7)	Relaxation of blood vessels -Suppresses inflammation (Patel et al., 2016)	Increases	-Reduces inflammatory response and reduces symptoms after SARS-CoV-2 infection

SARS-CoV-2, severe acute respiratory syndrome coronavirus 2; Th cell, T helper cell; NK cell, natural killer cell; DC cell, dendritic cell; pDC, plasmacytoid cell; mDC, myeloid cell; IFN-γ, interferon gamma; VEGF, vascular endothelial growth factor; IL-1α, interleukin 1 alpha; IL-1β, interleukin 1 beta; IL-2, interleukin 2; IL-4, interleukin 4; IL-6, interleukin 6; IL-7, interleukin 7; IL-8,interleukin 8; IL-10, interleukin 10; IL-12, interleukin 12; IL-13, interleukin 13; IL-17, interleukin 17; TNF, tumor necrosis factor; TGF-β, transforming growth factor-beta; LT-A, lymphotoxin-A; G-CSF, granulocyte-colony stimulating factor; GM-CSF, granulocyte-macrophage colony-stimulating factor; M-CSF, macrophage-colony stimulating factor; ACE2, angiotensin-converting enzyme 2; Ang 1-7, angiotensin-(1-7).

The CD3+/CD4+ and CD3+/CD8+ peripheral blood T lymphocyte and specific antiviral serum antibody counts were low in the pregnant patient (Chan et al., 2010). In addition to a decrease in the number of T cells in the blood during pregnancy, these cells are significantly less active when stimulated (Vazquez et al., 2015).

Transferring CD4+ T cell populations to T helper cell 2 (Th2) phenotypes during pregnancy (a response that promotes humoral rather than cellular immune responses) (Piccinni et al., 2000), reduced T helper cell 1 (Th1) reactivity may lead to reduced clearance of infected cells, increasing susceptibility to COVID-19 (Veenstra Van Nieuwenhoven et al., 2003). B cells are also affected by pregnancy, as their production is reduced during pregnancy (Medina et al., 1993).

Circulating natural killer (NK) cells decrease during pregnancy (Veenstra Van Nieuwenhoven et al., 2003). Since NK cells make up one fifth of the lymphocytes in the lung parenchyma, their role may provide a strong and extensive innate immune response to SARS-CoV-2 infection. Therefore, the decrease of NK cells is closely related to viral infection (Bozzano et al., 2021)..

Dendritic cells (DCs) are antigen-presenting cells that play a critical role in antiviral immunity (Liu, 2005). DCs are divided into two major types known as plasmacytoid cells (pDCs) and myeloid cells (mDCs) (Merad et al., 2013). Most studies have shown that the proportion of mDC/pDC is higher in pregnant women than in non-pregnant women (Darmochwal-Kolarz et al., 2003; Shin et al., 2009). During pregnancy, the production of interferon alpha (IFN-α) increases due to stimulation by toll-like receptors (TLRs), while the occurrence frequency of pDC decreases slightly, which may reduce the initiation of adaptive antiviral immune responses (Cordeau et al., 2012).

The phagocytic function of neutrophils and monocytes was significantly reduced in pregnant compared to non-pregnant women (Lampé et al., 2015).

The levels of interferon-γ (IFN-γ), vascular endothelial growth factor (VEGF), interleukin (IL)-1α, IL-1β, IL-6, IL-12, IL-17, IL-2, TNF-α (tumor necrosis factor α), and chemokines were decreased (Elenkov et al., 2001; Dashraath et al., 2020). On the contrary, IL-4, IL-10, IL-13, and transforming growth factor-β (TGF-β) levels were increased (Elenkov et al., 2001; Dashraath et al., 2020). Their changes during pregnancy are associated with a reduction in symptoms found in patients with COVID-19 (Pazos et al., 2012; Dashraath et al., 2020).

Circulating progesterone (P4) and steroid 17β-estradiol (E2) levels are elevated during pregnancy. The anti-inflammatory effects of E2 in innate immunity include inhibition of the production of pro-inflammatory cytokines such as IL-6 and IL-1β, tumor necrosis factor-α, monocytes, and macrophages which is a major factor in the COVID-19 cytokine storm (Chen R. et al., 2021) and powerful inhibition of monocyte chemoattractant protein-1 (MCP-1), thereby preventing innate immune cells, especially neutrophils and monocytes, from migrating to inflammatory areas. E2 stimulates CD4+ T helper cells to produce anti-inflammatory cytokines such as interleukin-4 (IL-4) and IL-10. In general, high E2 concentrations favor helper T cell type 2 (Th2) anti-inflammatory responses. E2 reduces production of interleukin-17 (IL-17) by pro-inflammatory T helper cell 17 (Th17 cells). E2 enhances the expansion of regulatory T cells (Treg), thereby promoting immune tolerance. E2 also stimulates the production of antibodies by B cells (Straub, 2007). P4 facilitates the CD4+ T-helper cell response from Th1 toward Th2 and the production of anti-inflammatory IL-4 and IL-10 cytokines (Szekeres-Bartho et al., 1996; Szekeres-Bartho and Wegmann, 1996).

## Activation and Response of the Immune System in Pregnant Women With COVID-19

**Figure 1:**
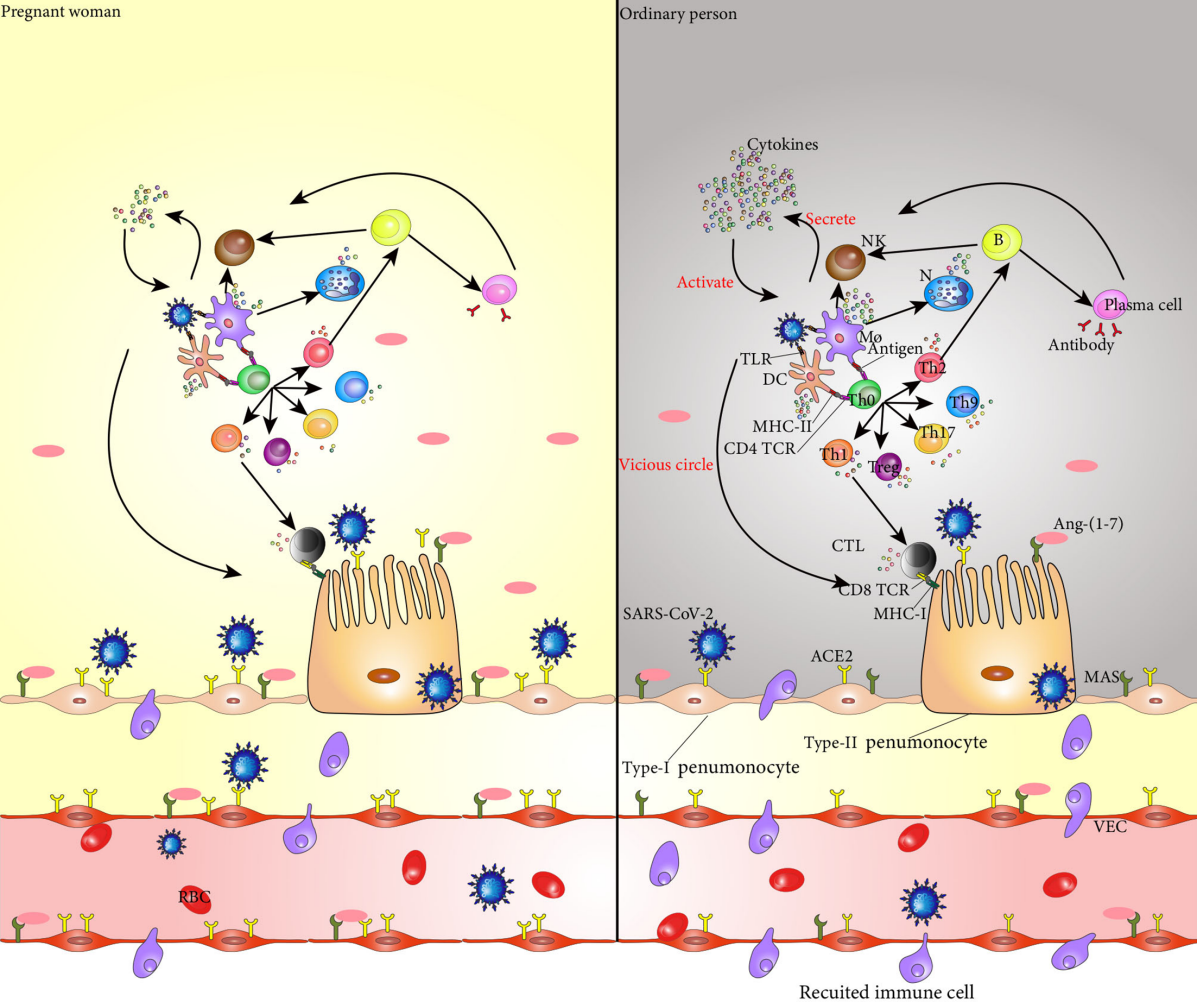
SARS-CoV-2 binds to ACE2 in the alveolar epithelial cells and invades these cells. Entry of SARS-CoV-2 into the body activates dendritic cells and macrophages, which secrete cytokines to activate NK cells. These NK cells kill the target cells infected by SARS-CoV-2. TLRs on dendritic cells and macrophages recognize viruses and secrete cytokines that mediate the inflammatory response. The CD4 TCR on the surface of Th0 cell membrane specifically binds to the antigen-peptide MHC II molecular complex on the surface of dendritic cells and macrophages, promoting the differentiation of Th0 cells into Th1, Th2, Treg, Th9, and Th17 cells, and the cytokines secreted by these cells promote the inflammatory response. Th1 cells secrete cytokines to activate CTL, and CD8 TCR on the surface of CTL recognizes and binds to MHC-I on the surface of cells infected with SARS-CoV-2 to kill target cells. Th2 cells secrete cytokines that assist the activation of B cells, which are activated into plasma cells that produce neutralizing antibodies to clear SARS-CoV-2. At the same time, B cells secrete cytokines to promote the killing activity of NK cells. The cytokines secreted by each immune cell in the immune response act on the immune cells themselves and promote their secretion of cytokines, forming a vicious cycle, finally resulting in a cytokine storm and aggravating the inflammatory response. In addition, Ang 1-7 produced by hydrolysis of ACE2 specifically binds to the PI3K/Akt and ERK signaling pathways regulated by MAS receptors on the cell surface, and plays an anti-inflammatory and cellular protective role. The increase of ACE2 levels in pregnant women makes invasion by SARS-CoV-2 easier; however, at the same time, the level of Ang 1-7 also increases, and the protective effect on the alveolar surface epithelial cells is stronger than that of non-pregnant women. During pregnancy, the immune system changes, and the cytokines secreted by dendritic cells and macrophages are reduced, and the activation of Th0 cells and NK cells is weakened, resulting in less cytokine. SARS-CoV-2, severe acute respiratory syndrome coronavirus 2; NK cell, natural killer cell; TLR, Toll-like receptors; Th0 cell, T helper cell 0; Th1 cell, T helper cell 2; Th2 cell, T helper cell 2; Treg, regulatory cells; Th9 cell, T helper cell 9; Th17 cell, T helper cell 17; CTL, cytotoxic T lymphocyte; MHC-I, major h*i*stocompat*i*b*i*l*i*ty complex-I; Ang-(1-7), angiotensin-(1-7); ACE2, angiotensin-converting enzyme 2; PI3K, phosphoinositide 3-kinase; ERK, extracellularsignal-regulated kinase;.

Th immune status of pregnant women with COVID-19 is unique which makes it easier for the virus to invade, and the rate of SARS-CoV-2 infection is higher in pregnant women than non-pregnant women. However, after SARS-CoV-2 invades a pregnant woman, her immune system confers a certain protective effect, and the serious consequences caused by the virus are likely reduced (Wei et al., 2020). Pregnant women hospitalized for COVID-19 are less likely to develop symptoms than non-pregnant women of similar age (Allotey et al., 2020) (**Figure 1**).

The angiotensin-converting enzyme 2 (ACE2) is a key receptor for coronavirus invasion. Similar to the way other coronaviruses invade the body, studies have shown that SARS-CoV-2 uses the spike glycoprotein (S) proteins to bind to its receptors on target cells (Wan et al., 2020; Zhou et al., 2020). ACE2 expression and activity are enhanced during pregnancy (Levy et al., 2008). Compared with non-pregnant women, pregnant women showed a two-fold increase in ACE2 receptor expression in different organs including the placenta, kidneys, and uterus (Brosnihan et al., 2004). This automatically makes pregnant women more susceptible to SARS-CoV-2 than non-pregnant women.

SARS-CoV-2 infects alveolar epithelial cells by recognizing the ACE2 receptor. Following cell invasion, the virus replicates in large quantities, which activates immune cells. Interestingly, ACE2 has a lung-protective effect (Ziegler et al., 2020), and elevated ACE2 levels in pregnant women make their lungs more protective than those of non-pregnant women, possibly reducing lung inflammation. In addition, angiopoietin 1-7 (Ang 1-7) produced by ACE2 degradation of Ang II was higher in pregnant women than in non-pregnant women, and the anti-inflammatory effect of phosphoinositide 3-kinase (PI3K)/Akt and extracellular signal-regulated kinase (ERK) signaling pathways regulated by MAS receptors specifically bound to Ang 1-7 was also stronger than that in non-pregnant women (Passos-Silva et al., 2013). This results in less severe COVID-19 symptoms in pregnant women than in non-pregnant women, fewer cytokine storms, and less progression towards acute respiratory distress syndrome (ARDS), multiple organ dysfunction, and death.

The pattern recognition receptors (PRRs) expressed by lung epithelial cells, macrophages, and DCs bind to damage-associated molecular patterns (DAMPs) produced by virus-infected epithelial cells and pathogen-associated molecular patterns (PAMPs) of the virus itself, which then activate nuclear factor-k-gene binding (NF-kB) and several mitogen-activated protein kinases (MAPKs), triggering a cascade of inflammatory responses and the release of chemokines. Inflammation is less severe in pregnant women and cytokine storms are less likely to occur. This is mainly caused by the following mechanisms. A physiological shift in pregnant women to a TH2 environment conducive to the expression of anti-inflammatory cytokines (IL-4 and IL-10). The levels of IFN-γ, VEGF, IL-1α, IL-1β, IL-6, IL-12, IL-17, and chemokines were decreased in pregnant woman, so the cascade of inflammatory cytokines was weaker than that of non-pregnant women. DCs activated by SARS-CoV-2 release IFN and IL-7 to promote immune inflammatory response and they trigger a direct antiviral response and manipulate the activation of NK cells and CTL (Hassanzadeh-Kiabi et al., 2017; Jamilloux et al., 2020). During pregnancy, the occurrence frequency of pDC decrease. Thus, DC-mediated inflammatory response was reduced in pregnant women. The phagocytic function of neutrophils and monocytes is significantly reduced during pregnancy and also releases fewer cytokines. NK cells help clear virus-infected cells through a variety of mechanisms including direct contact, cytokine or chemokine secretion, and indirect influence on lateral and downstream adaptive immune responses by affecting dendritic cells and T cells (Moretta, 2002; Mavilio et al., 2006; Vivier et al., 2011). The decrease of NK cells in pregnant women results in a significantly reduced inflammatory response in the body. The anti-inflammatory effects of E2 and P4 also play a role in reducing inflammatory response. As a result, the expression of pro-inflammatory and anti-inflammatory cytokines was relatively low in pregnant women with COVID-19. In particular, macrophage chemokines, referred to as major components of the cytokine storm during COVID-19 (Jafarzadeh et al., 2020), including macrophage inflammatory protein 1-alpha (MIP1α), CTACK, RANTES, eotaxin, growth-related oncogene alpha (GRO-α), and TNF were found at significantly low levels in pregnant patients. At the same time, basic fibroblast growth factor (FGF), leukemia inhibitory factor (LIF), granulocyte colony-stimulating factor (G-CSF), platelet-derived growth factor subunit B (PDGF-BB), and other growth factors were also significantly lower in COVID-19-positive pregnant women than pregnant women without COVID-19 (Chen G. et al., 2021).

CD4+ and CD8+ T cells not only eliminate the virus, but also stimulate hypermacrophage syndrome, eventually causing a cytokine storm in SARS-CoV-2 patients (Wang et al., 2020). The CD3+/CD4+ and CD3+/CD8+ peripheral blood T lymphocytes were low, which will reduce clearance of infected cells and the appearance of cytokine storm. Patients with COVID-19 show both a B cell immune response and a follicle-assisted T cell response (Thevarajan et al., 2020). In addition to producing antibodies, activated B cells also secrete IL-1, IL-6, IL-8, TNF, lymphotoxin-α (LT-A), G-CSF, granulocyte-macrophage colony-stimulating factor (GM-CSF), macrophage-colony stimulating factor (M-CSF), IL-7, and other cytokines that can aggravate the cytokine storm (Vazquez et al., 2015). Production of B cell lymphocytes is reduced during pregnancy, which alleviate the occurrence of cytokine storm.

A reduced inflammatory response also leads to delayed clearance of the virus and increased infection rates. This can lead to adverse obstetric outcomes.

## Adverse Pregnancy Outcomes due to COVID-19

Adverse obstetric outcomes of COVID-19 include miscarriage, intrauterine growth restriction, premature rupture of membranes, intrauterine/fetal distress, preeclampsia, vertical transmission, and preterm delivery (Dashraath et al., 2020; Schwartz, 2020) (**Table 2**).

**Table 2:** Immune effects of COVID-19 on pregnant women and adverse pregnancy outcomes.

Cell/Receptor/Cytokine	Function	Effects of COVID-19	Adverse pregnancy outcome
ECs	Release vasoactive factors to regulate vasoconstriction or relaxation Promotes inflammation (Mercier et al., 2017; Lin et al., 2019)	Dysfunction	Preeclampsia
Treg cells	Participates in the establishment of maternal-fetal immune tolerance Maintains immune homeostasis Treg/Th 17 balance promotes normal pregnancy (Kimura and Kishimoto, 2010; Lee J. H. et al., 2011; Gobert and Lafaille, 2012; Zhang et al., 2018)	Decreases Treg/Th 17↓	Abortion Preeclampsia Premature delivery
Th17 cell	Mediates tissue inflammation Treg/Th 17 balance promotes normal pregnancy (Kimura and Kishimoto, 2010; Zhang et al., 2018)	Increases Treg/Th 17↓	Abortion Preeclampsia Premature delivery
IFN-γ	Inhibition of angiogenesis Increases cytotoxic effects Promotes the differentiation of CD4+T cells to Th1 type and maintains the stability of Th1 phenotype Chemotaxis and activation of neutrophils, monocytes, and macrophages (Haep et al., 2015; Alspach et al., 2019)	Increases	Abortion Premature rupture of membranes
TNF	Enhances cytotoxicity Enhances the phagocytosis of neutrophils Damage to endothelial cells (Virdis et al., 2011; Brenner et al., 2015)	Increases	Abortion Premature rupture of membranes
IL-6	Stimulates the synthesis of APP in liver cells Stimulates the proliferation of activated B cells and secrete antibodies Stimulates T cell proliferation and CTL activation (Hirano, 2021)	Increases	Abortion Premature rupture of membranes
IL-8	Chemotactic neutrophils (Li Jeon et al., 2002)	Increases	Abortion Premature rupture of membranes
ACE2	Promotes angiotensin conversion -Key cell surface receptor that binds to SARS-CoV-2 (Levy et al., 2008; Jamilloux et al., 2020; Zhou et al., 2020)	Decreases	Preeclampsia
Ang-(1-7)	- Relaxation of blood vessels -Suppresses inflammation 10 (Patel et al., 2016)	Decreases	Preeclampsia
Blood coagulation factor	-Participates in blood clotting (Langarizadeh et al., 2021)	Increases	Thromboembolism

ECs, endothelial cells; Treg cells, regulatory cells; Th 17 cells, T helper cell 17; IFN-γ, interferon gamma; TNF, tumor necrosis factor; IL-6, interleukin 6; IL-8, interleukin 8; ACE2, angiotensin-converting enzyme 2; Ang 1-7), angiotensin-(1-7).

Endothelial cell dysfunction is the core mechanism that leads to preeclampsia in pregnant women (Burton et al., 2019). Endothelial dysfunction after SARS-CoV-2 infection is the key to progression of COVID-19, so pregnant women infected with COVID-19 are at an increased risk of developing preeclampsia (Bonaventura et al., 2021).

The delicate balance between Treg and Th17 cells mediates the mother’s tolerance to the fetus (Aluvihare et al., 2004; Lee J. H. et al., 2011). In patients with COVID-19, the level of Treg cells (CD3+CD4+CD25+ CD127low+) decreases (Wang et al., 2020; Qin et al., 2020), the level of Th17 cells (CCR6+ Th17) increases, and the ratio of Treg/Th17 cells decreases (Wu and Yang, 2020). Reduced Treg cell count and increased Th17 cell percentage are associated with pregnancy complications such as miscarriage, preeclampsia, and preterm delivery (Sasaki et al., 2007; Xiong et al., 2010; Fu et al., 2014; Koucký et al., 2014; Eghbal-Fard et al., 2019).

IFN-γ, TNF, IL-6, and IL-8 levels were significantly increased in COVID-19 patients (Huang et al., 2020), causing pregnant women with COVID-19 to be more prone to miscarriage and premature rupture of membranes. The secretion of TNF-α, IL-8, and IL-6 by macrophages in the process of inflammation can lead to abortion, premature rupture of membranes, and preterm labor (Lee S. Y. et al., 2011; Roncari et al., 2013; Li et al., 2015; Li et al., 2020).

SARS-CoV-2 not only binds to ACE2 but also causes it to be downregulated, so Ang 1-7) also decreases (Glowacka et al., 2010). Preeclampsia is associated with reduced plasma Ang 1-7) levels in the mother (Roncari et al., 2013). COVID-19 increases the risk of preeclampsia in pregnant women.

Patients with COVID-19 have a higher incidence of thromboembolic complications (Knight et al., 2020), and healthy pregnant women have higher levels of circulating clotting and fibrinolytic factors (such as fibrinolytic enzyme) (Di Renzo and Giardina, 2020). Alterations in clotting and fibrinolysis are thought to play an important role in the pathogenesis of preeclampsia (Schjetlein et al., 1997). Pregnant women with COVID-19 may have additive or co-operative risk factors for the onset of preeclampsia.

Studies have shown that that although vertical transmission of SARS-CoV-2 *in utero* is low, it is possible and appears to occur in a minority of pregnant women with COVID-19 in the third trimester (Fenizia et al., 2020; Kotlyar et al., 2021).

## Treatment

There are several treatments that hold promise for preventing and treating pregnant women with COVID-19 (**Table 3**).

**Table 3:** Treatment and prevention of COVID-19 in pregnant women. .

Therapeutic function	Therapeutic drug	Effect
Glucocorticoids		
	Dexamethasone	IL-6↓
Cytokine antagonists	Torzumab	Reduces inflammatory response and reduces symptoms after SARS-CoV-2 infection Delays virus clearance and increases infection rate
Anti-virus		
	Bamlanivimab	Accelerates the decline in the SARS-CoV-2 viral load (Dougan et al., 2021)
	Etesevimab	Accelerates the decline in the SARS-CoV-2 viral load (Dougan et al., 2021)
–	IFN-α	Blocks virus replication and transmission (Yang et al., 2021)
–	Remdesivir	Inhibits the replication of SARS coronavirus (Lamb, 2020)
Others	High-dose inhaled nitric oxide (160–200 ppm)	Antimicrobial effects against bacteria and viruses (including SARS-CoV)
–	Vit D	Regulates the imbalance of Treg/Th17
–	Magnesium sulfate	Prevents and treats epilepsy in preeclampsia
–	Aspirin	Prevents and treats epilepsy in preeclampsia
–	Metformin	Inhibits cytokine storm and prevents SARS-CoV-2 infection (Burwick et al., 2020)
Prevent		
Vaccine		Prevention of SARS-CoV-2 infection
Wear mask		Prevention of SARS-CoV-2 infection

IL-6, interleukin 6; IFN-α, interferon alpha; vit D, vitamin D; Treg cells, regulatory cells; Th 17 cells, T helper cell 17; SARS-CoV-2, severe acute respiratory syndrome coronavirus 2.

Bamlanivimab and etesevimab are recombinant human immunoglobulin G1 antibodies, which is a therapy that has proven to be more effective. A randomized clinical trial showed the treatment to be effective (Gottlieb et al., 2021). Phase III data on bamlanivimab plus etesevimab in COVID-19 patients showed that among non-hospitalized patients with mild-to-moderate COVID-19 disease, bamlanivimab plus etesevimab led to a lower incidence of COVID-19-related hospitalization and death than the placebo and accelerated the decline in the SARS-CoV-2 viral load (Dougan et al., 2021). The study population included high-risk groups such as immunosuppressed states, chronic diseases, immunodeficiency, and obesity. All these patients benefited from the treatment. Although there is still no data showing that it is safe for pregnant women, we believe this is a very promising treatment.

Torzumab is an IL-6 receptor antagonist and a meta-analysis of torzumab use and COVID-19 inpatient mortality showed that torzumab reduced all-cause mortality in COVID-19 inpatients within 28 days (Shankar-Hari et al., 2021). In addition, studies have shown that torzumab is effective in patients with severe COVID-19 during pregnancy and is a treatment option for COVID-19 during pregnancy (Naqvi et al., 2020).

There is some evidence that IFN can show anti-SARS-CoV-2 effect (Busnadiego et al., 2020; Monk et al., 2021), especially as inhaled nebulized interferon beta-1a (SNG001). Studies have shown that none of the patients who received IFN during pregnancy experienced stillbirth or delivered babies with severe malformations, and IFN did not significantly increase the risk of malformation, miscarriage, stillbirth, or preterm birth. Therefore, IFN is likely effective and safe in terms of COVID-19 during pregnancy (Yazdani Brojeni et al., 2012).

Remdesivir, a novel broad-spectrum antiviral nucleotide prodrug, has been shown to inhibit the replication of SARS coronavirus *in vitro*. Published results have suggested that the use of remdesivir may reduce clinical recovery time in patients with COVID-19 (Beigel et al., 2020). The manufacturer safety data of remdesivir indicate no reproductive developmental toxicity in animals at clinically relevant doses; furthermore, embryonic toxicity was only noted when systemically toxic doses were administered to female animals before conception (Maldarelli et al., 2020). Studies of the use of remdesivir did not document specific adverse outcomes in pregnant women (Mulangu et al., 2019; Burwick et al., 2020). These data provide patients and clinicians with reliable information about the safety of remdesivir in the treatment of pregnant women with COVID-19. Remdesivir is expected to be beneficial for COVID-19 therapy.

Dexamethasone is a common anti-inflammatory drug. It is known that glucocorticoids inhibit inflammation through non-genetic mechanisms, such as binding to glucocorticoid receptors on the T-cell membrane, resulting in disorder of receptor signals and immune response, and interaction with calcium and sodium across the cell membrane, leading to rapid resolution of inflammation (Langarizadeh et al., 2021). Hence, it may play a vital role in the treatment of COVID-19. The administration of prenatal corticosteroids to women at risk of preterm birth has been shown to have significant benefits in neonatal morbidity and mortality (Kemp et al., 2016), which make it reasonable for COVID-19 patients to use dexamethasone during pregnancy.

There is evidence for pregnant women with COVID-19 that appropriate use of magnesium sulfate, aspirin, metformin, and anticoagulants can help cure the disease and reduce adverse outcomes such as preeclampsia, premature birth, and miscarriage (Chen X. et al., 2020; D'Souza et al., 2021).

Vitamin D is an immunomodulatory hormone that has been established to be effective against a variety of upper respiratory tract infections. Vitamin D prevents excessive inflammatory response and accelerates the healing process in affected areas, primarily in lung tissue (Mohan et al., 2020). Moreover, Treg/Th17 imbalance can be corrected with vitamin D supplementation *in vivo (* Ji et al., 2019). The imbalance of Treg/Th17 in COVID-19 patients not only leads to uncontrolled cytokine release and increased inflammatory response in COVID-19 patients but also leads to adverse obstetric outcomes in pregnant patients. Vitamin D therefore holds promise as a complementary treatment.

High-dose inhaled nitric oxide (160–200 ppm) has shown antimicrobial effects against bacteria and viruses (including SARS-CoV), and is used as an adjunct treatment for ARDS and pulmonary hypertension. A case series of pregnant patients with severe COVID-19 treated with high-dose nitric oxide demonstrated improvement in hypoxemia and tachypnea with no adverse neonatal effects (Safaee Fakhr et al., 2020). Hence, it also holds promise as a complementary treatment.

We wish to highlight the need for close interdepartmental collaboration in caring for pregnant women presenting with COVID-19.

## Prevention

For the influenza virus, studies have shown significant reductions in low birth weight and preterm birth in vaccinated women compared to unvaccinated women (Heath et al., 2020), while other studies have not shown any association between vaccination and spontaneous abortion, preterm birth, birth defects, or small gestational age babies. Vaccination is one of the most promising preventive measures against COVID-19. Because pregnant women are more susceptible to SARS-CoV-2, vaccination during pregnancy is the most optimal strategy for preventing maternal and neonatal disease (Andreani et al., 2020; Heath et al., 2020). A preliminary finding of mRNA safety of COVID-19 vaccine in pregnant women showed that vaccination is safe in the third trimester of pregnancy (Shimabukuro et al., 2021). In addition to vaccination protecting women against COVID-19 and its complications during pregnancy, emerging evidence has shown transplacental transfer of SARS-CoV-2 antibodies after maternal COVID-19 vaccination during the third trimester, which suggests that maternal vaccination might provide some level of protection to the neonate (Gill and Jones, 2021; Gray et al., 2021; Rottenstreich et al., 2021). To date, there is still little information on the efficacy and safety of vaccinating pregnant women. More extensive research is required regarding vaccinating pregnant women.

Studies (Cheng et al., 2021) have shown that wearing a surgical mask can be a very cost-effective method to prevent the spread of virus. Because the vaccine does not provide 100% protection against COVID-19, mask-wearing is especially important for pregnant women who are more vulnerable to infection.

## Conclusion

COVID-19-related immune system changes in pregnant women involve multiple cytokines, cells, and receptors. The unique immune status of pregnant women makes them more susceptible to SARS-CoV-2 invasion. However, after SARS-CoV-2 invades a pregnant woman, the immune system of the body has a certain protective effect, which reduces the serious consequences caused by the virus. However, changes in the immune system caused by the virus can also lead to poor pregnancy outcomes. Therefore, it is of great significance to accurately identify COVID-19 inflammatory pathways and therapeutic targets in pregnant women. The efficacy of the current COVID-19 treatment for pregnant women is still not satisfactory. Many clinical studies of drugs and vaccines have excluded pregnant women. The treatment of pregnant women has become a challenging problem, and more studies are needed to address these issues.

## Author Contributions 

HS and HY conceived and designed the study. RC, SZ, and SS performed the literature search and drafted the manuscript. All authors contributed to the article and approved the submitted version.

## Funding

This project was supported by Medical Affairs Department and Scientific Research department of Guangdong Provincial People’s Hospital and the Scientific Research Initial Funding of Guangdong Provincial People’s Hospital awarded to HS (KJ012019529).

## Conflict of Interest

The authors declare that the research was conducted in the absence of any commercial or financial relationships that could be construed as a potential conflict of interest.

## Publisher’s Note

All claims expressed in this article are solely those of the authors and do not necessarily represent those of their affiliated organizations, or those of the publisher, the editors and the reviewers. Any product that may be evaluated in this article, or claim that may be made by its manufacturer, is not guaranteed or endorsed by the publisher.

## References

[B1] Allotey J. Stallings E. Bonet M. Yap M. Chatterjee S. Kew T.. (2020). Clinical Manifestations, Risk Factors, and Maternal and Perinatal Outcomes of Coronavirus Disease 2019 in Pregnancy: Living Systematic Review and Meta-Analysis. BMJ (Clinical Res. ed.) 370, m3320. 10.1136/bmj.m3320 PMC7459193 32873575

[B2] Alspach E. Lussier D. M. Schreiber R. D. (2019). Interferon γ and Its Important Roles in Promoting and Inhibiting Spontaneous and Therapeutic Cancer Immunity. Csh Perspect. Biol. 11 (3), a028480. 10.1101/cshperspect.a028480 PMC6396335 29661791

[B3] Aluvihare V. R. Kallikourdis M. Betz A. G. (2004). Regulatory T Cells Mediate Maternal Tolerance to the Fetus. Nat. Immunol. 5 (3), 266–271. 10.1038/ni1037 14758358

[B4] Amatya N. Garg A. V. Gaffen S. L. (2017). IL-17 Signaling: The Yin and the Yang. Trends Immunol. 38 (5), 310–322. 10.1016/j.it.2017.01.006 28254169 PMC5411326

[B5] Andreani J. Le Bideau M. Duflot I. Jardot P. Rolland C. Boxberger M.. (2020). *In Vitro* Testing of Combined Hydroxychloroquine and Azithromycin on SARS-CoV-2 Shows Synergistic Effect. Microb. Pathogenesis 145, 104228. 10.1016/j.micpath.2020.104228 PMC7182748 32344177

[B6] Andrikopoulou M. Madden N. Wen T. Aubey J. Aziz J. Aleha C. D.. (2020). Symptoms and Critical Illness Among Obstetric Patients With Coronavirus Disease 2019 (COVID-19) Infection. Obstet. Gynecol. 136 (2), 291–299. 10.1097/AOG.0000000000003996 32459701

[B7] Beigel J. H. Tomashek K. M. Dodd L. E. Mehta A. K. Zingman B. S. Kalil A. C.. (2020). Remdesivir for the Treatment of Covid-19 - Final Report. New Engl. J. Med. 383 (19), 1813–1826. 10.1056/NEJMoa2007764 32445440 PMC7262788

[B8] Bonaventura A. Vecchié A. Dagna L. Martinod K. Dixon D. L. Tassell B. W. V.. (2021). Endothelial Dysfunction and Immunothrombosis as Key Pathogenic Mechanisms in COVID-19. Nat. Rev. Immunol. 21 (5), 319–329. 10.1038/s41577-021-00536-9 33824483 PMC8023349

[B9] Bozzano F. Dentone C. Perrone C. Biagio A. D. Fenoglio D. Parodi A.. (2021). Extensive Activation, Tissue Trafficking, Turnover and Functional Impairment of NK Cells in COVID-19 Patients at Disease Onset Associates With Subsequent Disease Severity. PloS Pathog. 17 (4), e1009448. 10.1371/journal.ppat.1009448 33861802 PMC8081333

[B10] Brenner D. Blaser H. Mak T. W. (2015). Regulation of Tumour Necrosis Factor Signalling: Live or Let Die. Nat. Rev. Immunol. 15 (6), 362–374. 10.1038/nri3834 26008591

[B11] Brosnihan K. B. Neves L. A. Anton L. Joyner J. Valdes G. Merrill D. C. (2004). Enhanced Expression of Ang-(1-7) During Pregnancy. Braz. J. Med. Biol. Res. = Rev. Bras. Pesquisas Medicas e Biologicas. 37 (8), 1255–1262. 10.1590/S0100-879X2004000800017 15273828

[B12] Burton G. J. Redman C. W. Roberts J. M. Moffett A. (2019). Pre-Eclampsia: Pathophysiology and Clinical Implications. BMJ (Clinical Res. ed.) 366, l2381. 10.1136/bmj.l2381 31307997

[B13] Burwick R. M. Yawetz S. Stephenson K. E. Collier A. Y. Sen P. Blackburn B. G.. (2020). Compassionate Use of Remdesivir in Pregnant Women With Severe Covid-19. Clin. Infect. Dis. an Off. Publ. Infect. Dis. Soc. A. 1–9. 10.1093/cid/ciaa1466 PMC7797739 33031500

[B14] Busnadiego I. Fernbach S. Pohl M. O. Karakus U. Huber M. Trkola A. (2020). Antiviral Activity of Type I, II, and III Interferons Counterbalances ACE2 Inducibility and Restricts SARS-CoV-2. Mbio 11 (5), e01928-20. 10.1128/mBio.01928-20 PMC7484541 32913009

[B15] Chan K. H. Zhang A. J. To K. K. Chan C. C. S. Poon V. K. K. Guo K.. (2010). Wild Type and Mutant 2009 Pandemic Influenza A (H1N1) Viruses Cause More Severe Disease and Higher Mortality in Pregnant BALB/c Mice. PloS One 5 (10), e13757. 10.1371/journal.pone.0013757 21060798 PMC2966430

[B16] Cheng Y. Ma N. Witt C. Rapp S. Wild P. S. Andreae M. O.. (2021). Face Masks Effectively Limit the Probability of SARS-CoV-2 Transmission. Sci. (New York N.Y.) 372 (6549), 1439–1443. 10.1126/science.abg6296 PMC8168616 34016743

[B17] Chen X. Guo H. Qiu L. Zhang C. Deng Q. Leng Q. (2020). Immunomodulatory and Antiviral Activity of Metformin and Its Potential Implications in Treating Coronavirus Disease 2019 and Lung Injury. Front. Immunol. 11, 2056. 10.3389/fimmu.2020.02056 32973814 PMC7461864

[B18] Chen H. Guo J. Wang C. Luo F. Yu X. Zhang W.. (2020). Clinical Characteristics and Intrauterine Vertical Transmission Potential of COVID-19 Infection in Nine Pregnant Women: A Retrospective Review of Medical Records. Lancet (London England) 395 (10226), 809–815. 10.1016/S0140-6736(20)30360-3 PMC7159281 32151335

[B19] Chen R. Lan Z. Ye J. Pang L. Liu Y. Wu W.. (2021). Cytokine Storm: The Primary Determinant for the Pathophysiological Evolution of COVID-19 Deterioration. Front. Immunol. 12, 589095. 10.3389/fimmu.2021.589095 33995341 PMC8115911

[B20] Chen G. Liao Q. Ai J. Yang B. Bai H. Chen J.. (2021). Immune Response to COVID-19 During Pregnancy. Front. Immunol. 12, 675476. 10.3389/fimmu.2021.675476 34012458 PMC8126657

[B21] Chen J. Zhao Y. Chu X. Lu Y. Wang S. Yi Q. (2016). Dectin-1-Activated Dendritic Cells: A Potent Th9 Cell Inducer for Tumor Immunotherapy. Oncoimmunology 5 (11), e1238558. 10.1080/2162402X.2016.1238558 27999759 PMC5139651

[B22] Cordeau M. Herblot S. Charrier E. Audibert F. Cordeiro P. Harnois M.. (2012). Defects in CD54 and CD86 Up-Regulation by Plasmacytoid Dendritic Cells During Pregnancy. Immunol. Invest. 41 (5), 497–506. 10.3109/08820139.2012.682243 22594887

[B23] D'Souza R. Ashraf R. Rowe H. Zipursky J. Clarfield L. Maxwell C.. (2021). Pregnancy and COVID-19: Pharmacologic Considerations. Ultrasound Obstet. Gynecol. Off. J. Int. Soc. Ultrasound Obstet. Gynecol. 57 (2), 195–203. 10.1002/uog.23116 PMC7537532 32959455

[B24] Darmochwal-Kolarz D. Rolinski J. Tabarkiewicz J. Leszczynska-Gorzelak B. Buczkowski J. Wojas K.. (2003). Myeloid and Lymphoid Dendritic Cells in Normal Pregnancy and Pre-Eclampsia. Clin. Exp. Immunol. 132 (2), 339–344. 10.1046/j.1365-2249.2003.02136.x 12699426 PMC1808701

[B25] Dashraath P. Wong J. Lim M. Lim L. Li S. Biswas A.. (2020). Coronavirus Disease 2019 (COVID-19) Pandemic and Pregnancy. Am. J. Obstet. Gynecol. 222 (6), 521–531. 10.1016/j.ajog.2020.03.021 32217113 PMC7270569

[B26] Di Renzo G. C. Giardina I. (2020). Coronavirus Disease 2019 in Pregnancy: Consider Thromboembolic Disorders and Thromboprophylaxis. Am. J. Obstet. Gynecol. 223 (1), 135. 10.1016/j.ajog.2020.04.017 PMC7175884 32333857

[B27] Dougan M. Nirula A. Azizad M. Mocherla B. Gottlieb R. L. Chen P.. (2021). Bamlanivimab Plus Etesevimab in Mild or Moderate Covid-19. New Engl. J. Med. 2102685. 10.1056/NEJMoa2102685 PMC8314785 34260849

[B28] Eghbal-Fard S. Yousefi M. Heydarlou H. Ahmadi M. Taghavi S. Movasaghpour A.. (2019). The Imbalance of Th17/Treg Axis Involved in the Pathogenesis of Preeclampsia. J. Cell Physiol. 234 (4), 5106–5116. 10.1002/jcp.27315 30277561

[B29] Elenkov I. J. Wilder R. L. Bakalov V. K. Link A. A. Dimitrov M. A. Fisher S.. (2001). IL-12, TNF-Alpha, and Hormonal Changes During Late Pregnancy and Early Postpartum: Implications for Autoimmune Disease Activity During These Times. J. Clin. Endocrinol. Metab. 86 (10), 4933–4938. 10.1210/jcem.86.10.7905 11600565

[B30] Fenizia C. Biasin M. Cetin I. Vergani P. Mileto D. Spinillo A.. (2020). Analysis of SARS-CoV-2 Vertical Transmission During Pregnancy. Nat. Commun. 11 (1), 5128. 10.1038/s41467-020-18933-4 33046695 PMC7552412

[B31] Franke F. Kirchenbaum G. A. Kuerten S. Lehmann P. V. (2020). IL-21 in Conjunction With Anti-CD40 and IL-4 Constitutes a Potent Polyclonal B Cell Stimulator for Monitoring Antigen-Specific Memory B Cells. Cells-Basel 9 (2), 433. 10.3390/cells9020433 PMC7072853 32069813

[B32] Furue K. Ito T. Tsuji G. Ulzii D. Vu Y. H. Kido-Nakahara M.. (2019). The IL-13-OVOL1-FLG Axis in Atopic Dermatitis. Immunology 158 (4), 281–286. 10.1111/imm.13120 31509236 PMC6856930

[B33] Fu B. Tian Z. Wei H. (2014). TH17 Cells in Human Recurrent Pregnancy Loss and Pre-Eclampsia. Cell Mol. Immunol. 11 (6), 564–570. 10.1038/cmi.2014.54 25027967 PMC4220838

[B34] Gao Y. J. Ye L. Zhang J. S. Yin Y. X. Liu M. Yu H. B.. (2020). Clinical Features and Outcomes of Pregnant Women With COVID-19: A Systematic Review and Meta-Analysis. BMC Infect. Dis. 20 (1), 564. 10.1186/s12879-020-05274-2 32746801 PMC7396931

[B35] Gill L. Jones C. W. (2021). Severe Acute Respiratory Syndrome Coronavirus 2 (SARS-CoV-2) Antibodies in Neonatal Cord Blood After Vaccination in Pregnancy. Obstet. Gynecol. 137 (5), 894–896. 10.1097/AOG.0000000000004367 33684922

[B36] Glowacka I. Bertram S. Herzog P. Pfefferle S. Steffen I. Muench M. O.. (2010). Differential Downregulation of ACE2 by the Spike Proteins of Severe Acute Respiratory Syndrome Coronavirus and Human Coronavirus NL63. J. Virol. 84 (2), 1198–1205. 10.1128/JVI.01248-09 19864379 PMC2798380

[B37] Gobert M. Lafaille J. J. (2012). Maternal-Fetal Immune Tolerance, Block by Block. Cell 150 (1), 7–9. 10.1016/j.cell.2012.06.020 22770210 PMC4061910

[B38] Gottlieb R. L. Nirula A. Chen P. Boscia J. Heller B. Morris J.. (2021). Effect of Bamlanivimab as Monotherapy or in Combination With Etesevimab on Viral Load in Patients With Mild to Moderate COVID-19: A Randomized Clinical Trial. JAMA 325 (7), 632–644. 10.1001/jama.2021.0202 33475701 PMC7821080

[B39] Gray K. J. Bordt E. A. Atyeo C. Deriso E. Akinwunmi B. Young N.. (2021). COVID-19 Vaccine Response in Pregnant and Lactating Women: A Cohort Study. medRxiv Preprint Server Health Sci. 10.1101/2021.03.07.21253094 PMC7997025 33775692

[B40] Grunwell J. R. Stephenson S. T. Tirouvanziam R. Brown L. Brown M. R. Fitzpatrick A. M. (2019). Children With Neutrophil-Predominant Severe Asthma Have Proinflammatory Neutrophils With Enhanced Survival and Impaired Clearance. J. Allergy Clin. Immunol. In Pract. 7 (2), 516–525. 10.1016/j.jaip.2018.08.024 30193935 PMC6363859

[B41] Guilliams M. Mildner A. Yona S. (2018). Developmental and Functional Heterogeneity of Monocytes. Immunity 49 (4), 595–613. 10.1016/j.immuni.2018.10.005 30332628

[B42] Haep L. Britzen-Laurent N. Weber T. G. Naschberger E. Schaefer A. Kremmer E.. (2015). Interferon Gamma Counteracts the Angiogenic Switch and Induces Vascular Permeability in Dextran Sulfate Sodium Colitis in Mice. Inflammation Bowel Dis. 21 (10), 2360–2371. 10.1097/MIB.0000000000000490 26164664

[B43] Hart P. H. Bonder C. S. Balogh J. Dickensheets H. L. Donnelly R. P. Finlay-Jones J. J. (1999). Differential Responses of Human Monocytes and Macrophages to IL-4 and IL-13. J. Leukocyte Biol. 66 (4), 575–578. 10.1002/jlb.66.4.575 10534111

[B44] Hassanzadeh-Kiabi N. Yáñez A. Dang I. Martins G. A. Underhill D. M. Goodridge H. S. (2017). Autocrine Type I IFN Signaling in Dendritic Cells Stimulated With Fungal β-Glucans or Lipopolysaccharide Promotes CD8 T Cell Activation. J. Immunol. (Baltimore Md. 1950) 198 (1), 375–382. 10.4049/jimmunol.1601143 PMC5173402 27872213

[B45] Heath P. T. Le Doare K. Khalil A. (2020). Inclusion of Pregnant Women in COVID-19 Vaccine Development. Lancet Infect. Diseases 20 (9), 1007–1008. 10.1016/S1473-3099(20)30638-1 32795409 PMC7831663

[B46] Hirano T. (2021). IL-6 in Inflammation, Autoimmunity and Cancer. Int. Immunol. 33 (3), 127–148. 10.1093/intimm/dxaa078 33337480 PMC7799025

[B47] Holmes K. Roberts O. L. Thomas A. M. Cross M. J. (2007). Vascular Endothelial Growth Factor Receptor-2: Structure, Function, Intracellular Signalling and Therapeutic Inhibition. Cell Signal. 19 (10), 2003–2012. 10.1016/j.cellsig.2007.05.013 17658244

[B48] Horkova V. Drobek A. Mueller D. Gubser C. Niederlova V. Wyss L.. (2020). Dynamics of the Coreceptor-LCK Interactions During T Cell Development Shape the Self-Reactivity of Peripheral CD4 and CD8 T Cells. Cell Rep. 30 (5), 1504–1514. 10.1016/j.celrep.2020.01.008 32023465 PMC7003063

[B49] Huang C. Wang Y. Li X. Ren L. Zhao J. Hu Y.. (2020). Clinical Features of Patients Infected With 2019 Novel Coronavirus in Wuhan, China. Lancet (London England) 395 (10223), 497–506. 10.1016/S0140-6736(20)30183-5 PMC7159299 31986264

[B50] Huntington N. D. Cursons J. Rautela J. (2020). The Cancer-Natural Killer Cell Immunity Cycle. Nat. Rev. Cancer 20 (8), 437–454. 10.1038/s41568-020-0272-z 32581320

[B51] Islam M. M. Poly T. N. Walther B. A. Yang H. C. Wang C. W. Hsieh W. S.. (2020). Clinical Characteristics and Neonatal Outcomes of Pregnant Patients With COVID-19: A Systematic Review. Front. Med. 7, 573468. 10.3389/fmed.2020.573468 PMC7772992 33392213

[B52] Jafarzadeh A. Chauhan P. Saha B. Jafarzadeh S. Nemati M. (2020). Contribution of Monocytes and Macrophages to the Local Tissue Inflammation and Cytokine Storm in COVID-19: Lessons From SARS and MERS, and Potential Therapeutic Interventions. Life Sci. 257, 118102. 10.1016/j.lfs.2020.118102 32687918 PMC7367812

[B53] Jaillon S. Ponzetta A. Di Mitri D. Santoni A. Bonecchi R. Mantovani A. (2020). Neutrophil Diversity and Plasticity in Tumour Progression and Therapy. Nat. Rev. Cancer 20 (9), 485–503. 10.1038/s41568-020-0281-y 32694624

[B54] Jamilloux Y. Henry T. Belot A. Viel S. Fautr M. Jammal T. E.. (2020). Should We Stimulate or Suppress Immune Responses in COVID-19? Cytokine and Anti-Cytokine Interventions. Autoimmun. Rev. 19 (7), 102567. 10.1016/j.autrev.2020.102567 32376392 PMC7196557

[B55] Ji J. Zhai H. Zhou H. Song S. Mor G. Liao A. (2019). The Role and Mechanism of Vitamin D-Mediated Regulation of Treg/Th17 Balance in Recurrent Pregnancy Loss. Am. J. Reprod. Immunol. (New York N.Y. 1989) 81 (6), e13112. 10.1111/aji.13112 30903715

[B56] Junttila I. S. (2018). Tuning the Cytokine Responses: An Update on Interleukin (IL)-4 and IL-13 Receptor Complexes. Front. Immunol. 9, 888. 10.3389/fimmu.2018.00888 29930549 PMC6001902

[B57] Kemp M. W. Newnham J. P. Challis J. G. Jobe A. H. Stock S. J. (2016). The Clinical Use of Corticosteroids in Pregnancy. Hum. Reprod. Update 22 (2), 240–259. 10.1093/humupd/dmv047 26590298

[B58] Kimura A. Kishimoto T. (2010). IL-6: Regulator of Treg/Th17 Balance. Eur. J. Immunol. 40 (7), 1830–1835. 10.1002/eji.201040391 20583029

[B59] Knight M. Bunch K. Vousden N. Morris E. Simpson N. Gale C.. (2020). Characteristics and Outcomes of Pregnant Women Admitted to Hospital With Confirmed SARS-CoV-2 Infection in UK: National Population Based Cohort Study. BMJ (Clinical Res. ed.) 369, m2107. 10.1136/bmj.m2107 PMC7277610 32513659

[B60] Kotlyar A. M. Grechukhina O. Chen A. Popkhadze S. Grimshaw A. Tal O.. (2021). Vertical Transmission of Coronavirus Disease 2019: A Systematic Review and Meta-Analysis. Am. J. Obstet. Gynecol. 224 (1), 35–53. 10.1016/j.ajog.2020.07.049 32739398 PMC7392880

[B61] Koucký M. Malíčková K. Cindrová-Davies T. Germanova A. Parizek A. Kalousova M.. (2014). Low Levels of Circulating T-Regulatory Lymphocytes and Short Cervical Length are Associated With Preterm Labor. J. Reprod. Immunol. 106, 110–117. 10.1016/j.jri.2014.04.001 24855050

[B62] Lamb Y. N. (2020). Remdesivir: First Approval. Drugs 80 (13), 1355–1363. 10.1007/s40265-020-01378-w 32870481 PMC7459246

[B63] Lampé R. Kövér Á. Szűcs S. Pal L. Arnyas E. Adany R.. (2015). Phagocytic Index of Neutrophil Granulocytes and Monocytes in Healthy and Preeclamptic Pregnancy. J. Reprod. Immunol. 107, 26–30. 10.1016/j.jri.2014.11.001 25534923

[B64] Langarizadeh M. A. Ranjbar Tavakoli M. Abiri A. Ghasempour A. Rezaei M. (2021). Ameri A. A Review on Function and Side Effects of Systemic Corticosteroids Used in High-Grade COVID-19 to Prevent Cytokine Storms. Excli. J. 20, 339–365. 10.17179/excli2020-3196 33746666 PMC7975631

[B65] Laurence A. Tato C. M. Davidson T. S. Kanno Y. Chen Z. Yao Z.. (2007). Interleukin-2 Signaling *via* STAT5 Constrains T Helper 17 Cell Generation. Immunity 26 (3), 371–381. 10.1016/j.immuni.2007.02.009 17363300

[B66] Lee S. Y. Buhimschi I. A. Dulay A. T. Ali U. A. Zhao G. Abdel-Razeq S. S.. (2011). IL-6 Trans-Signaling System in Intra-Amniotic Inflammation, Preterm Birth, and Preterm Premature Rupture of the Membranes. J. Immunol. (Baltimore Md. 1950) 186 (5), 3226–3236. 10.4049/jimmunol.1003587 PMC3800180 21282511

[B67] Lee J. H. Ulrich B. Cho J. Park J. Kim C. H. (2011). Progesterone Promotes Differentiation of Human Cord Blood Fetal T Cells Into T Regulatory Cells But Suppresses Their Differentiation Into Th17 Cells. J. Immunol. (Baltimore Md. 1950) 187 (4), 1778–1787. 10.4049/jimmunol.1003919 PMC3155957 21768398

[B68] Levy A. Yagil Y. Bursztyn M. Barkalifa R. Scharf S. Yagil C. (2008). ACE2 Expression and Activity are Enhanced During Pregnancy. Am. J. Physiol. Regul. Integr. Comp. Physiol. 295 (6), R1953–R1961. 10.1152/ajpregu.90592.2008 18945956

[B69] Li Jeon N. Baskaran H. Dertinger S. K. Whitesides G. M. Van de Water L. Toner M. (2002). Neutrophil Chemotaxis in Linear and Complex Gradients of Interleukin-8 Formed in a Microfabricated Device. Nat. Biotechnol. 20 (8), 826–830. 10.1038/nbt712 12091913

[B70] Lin Q. Zhao L. Jing R. Trexler C. Wang H. Li Y.. (2019). Inositol 1,4,5-Trisphosphate Receptors in Endothelial Cells Play an Essential Role in Vasodilation and Blood Pressure Regulation. J. Am. Heart Assoc. 8 (4), e11704. 10.1161/JAHA.118.011704 PMC6405661 30755057

[B71] Li Z. Y. Song Z. H. Meng C. Y. Yang D. D. Yang Y. Peng J. P. (2015). IFN-γ Modulates Ly-49 Receptors on NK Cells in IFN-γ-Induced Pregnancy Failure. Sci. Rep. Uk. 5, 18159. 10.1038/srep18159 PMC4676035 26655673

[B72] Liu Y. J. (2005). IPC: Professional Type 1 Interferon-Producing Cells and Plasmacytoid Dendritic Cell Precursors. Annu. Rev. Immunol. 23, 275–306. 10.1146/annurev.immunol.23.021704.115633 15771572

[B73] Li W. Zhao X. Li S. Chen X. Cui H. Chang Y.. (2020). Upregulation of TNF-α and IL-6 Induces Preterm Premature Rupture of Membranes by Activation of ADAMTS-9 in Embryonic Membrane Cells. Life Sci. 260, 118237. 10.1016/j.lfs.2020.118237 32781068

[B74] Maldarelli G. A. Savage M. Mazur S. Oxford-Horrey C. Salvatore M. Marks K. M. (2020). Remdesivir Treatment for Severe COVID-19 in Third-Trimester Pregnancy: Case Report and Management Discussion. Open Forum Infect. Di. 7 (9), a345. 10.1093/ofid/ofaa345 PMC7478602 32934969

[B75] Mantovani A. Dinarello C. A. Molgora M. Garlanda C. (2019). Interleukin-1 and Related Cytokines in the Regulation of Inflammation and Immunity. Immunity 50 (4), 778–795. 10.1016/j.immuni.2019.03.012 30995499 PMC7174020

[B76] Mavilio D. Lombardo G. Kinter A. Fogli M. Sala A. L. Ortolano S.. (2006). Characterization of the Defective Interaction Between a Subset of Natural Killer Cells and Dendritic Cells in HIV-1 Infection. J. Exp. Med. 203 (10), 2339–2350. 10.1084/jem.20060894 17000867 PMC2118111

[B77] Medina K. L. Smithson G. Kincade P. W. (1993). Suppression of B Lymphopoiesis During Normal Pregnancy. J. Exp. Med. 178 (5), 1507–1515. 10.1084/jem.178.5.1507 8228804 PMC2191236

[B78] Merad M. Sathe P. Helft J. Miller J. Mortha A. (2013). The Dendritic Cell Lineage: Ontogeny and Function of Dendritic Cells and Their Subsets in the Steady State and the Inflamed Setting. Annu. Rev. Immunol. 31, 563–604. 10.1146/annurev-immunol-020711-074950 23516985 PMC3853342

[B79] Mercier O. Arthur Ataam J. Langer N. B. Dorfmuller P. Lamrani L. Lecerf F.. (2017). Abnormal Pulmonary Endothelial Cells may Underlie the Enigmatic Pathogenesis of Chronic Thromboembolic Pulmonary Hypertension. J. Heart Lung Transplant. Off. Publ. Int. Soc. Heart Transpl. 36 (3), 305–314. 10.1016/j.healun.2016.08.012 27793518

[B80] Mesner D. Hotter D. Kirchhoff F. Jolly C. (2020). Loss of Nef-Mediated CD3 Down-Regulation in the HIV-1 Lineage Increases Viral Infectivity and Spread. P Natl. Acad. Sci. U.S.A. 117 (13), 7382–7391. 10.1073/pnas.1921135117 PMC7132320 32179688

[B81] Mohan M. Cherian J. J. Sharma A. (2020). Exploring Links Between Vitamin D Deficiency and COVID-19. PloS Pathog. 16 (9), e1008874. 10.1371/journal.ppat.1008874 32946517 PMC7500624

[B82] Monk P. D. Marsden R. J. Tear V. J. Brookes J. Batten T. N. Mankowski M.. (2021). Safety and Efficacy of Inhaled Nebulised Interferon Beta-1a (SNG001) for Treatment of SARS-CoV-2 Infection: A Randomised, Double-Blind, Placebo-Controlled, Phase 2 Trial. Lancet Respir. Med. 9 (2), 196–206. 10.1016/S2213-2600(20)30511-7 33189161 PMC7836724

[B83] Moretta A. (2002). Natural Killer Cells and Dendritic Cells: Rendezvous in Abused Tissues. Nat. Rev. Immunol. 2 (12), 957–964. 10.1038/nri956 12461568

[B84] Mulangu S. Dodd L. E. Davey R. T. Mbaya O. T. Proschan M. Mukadi D. (2019). A Randomized, Controlled Trial of Ebola Virus Disease Therapeutics. New Engl. J. Med. 381 (24), 2293–2303. 10.1056/NEJMoa1910993 31774950 PMC10680050

[B85] Naqvi M. Zakowski P. Glucksman L. Smithson S. Burwick R. M. (2020). Tocilizumab and Remdesivir in a Pregnant Patient With Coronavirus Disease 2019 (COVID-19). Obstet. Gynecol. 136 (5), 1025–1029. 10.1097/AOG.0000000000004050 32618794

[B86] Passos-Silva D. G. Verano-Braga T. Santos R. A. (2013). Angiotensin-(1-7): Beyond the Cardio-Renal Actions. Clin. Sci. (London Engl. 1979). 124 (7), 443–456. 10.1042/CS20120461 23249272

[B87] Patel V. B. Zhong J. C. Grant M. B. Oudit G. Y. (2016). Role of the ACE2/Angiotensin 1-7 Axis of the Renin-Angiotensin System in Heart Failure. Circ. Res. 118 (8), 1313–1326. 10.1161/CIRCRESAHA.116.307708 27081112 PMC4939482

[B88] Pazos M. Sperling R. S. Moran T. M. Kraus T. A. (2012). The Influence of Pregnancy on Systemic Immunity. Immunol. Res. 54, 254–261. 10.1007/s12026-012-8303-9 22447351 PMC7091327

[B89] Piccinni M. P. Maggi E. Romagnani S. (2000). Role of Hormone-Controlled T-Cell Cytokines in the Maintenance of Pregnancy. Biochem. Soc. T. 28 (2), 212–215. 10.1042/bst0280212 10816130

[B90] Qin C. Zhou L. Hu Z. Zhang S. Yang S. Tao Y.. (2020). Dysregulation of Immune Response in Patients With Coronavirus 2019 (COVID-19) in Wuhan, China. Clin. Infect. Dis. An Off. Publ. Infect. Dis. Soc. A. 71 (15), 762–768. 10.1093/cid/ciaa248 PMC7108125 32161940

[B91] Roncari D. Politch J. A. Sonalkar S. Finneseth M. Borgatta L. (2013). Inflammation or Infection at the Time of Second Trimester Induced Abortion. Contraception 87 (1), 67–70. 10.1016/j.contraception.2012.09.016 23102591

[B92] Rottenstreich A. Zarbiv G. Oiknine-Djian E. Zigron R. Wolf D. G. Porat S. (2021). Efficient Maternofetal Transplacental Transfer of Anti- SARS-CoV-2 Spike Antibodies After Antenatal SARS-CoV-2 BNT162b2 mRNA Vaccination. Clin. Infect. Dis. An Off. Publ. Infect. Dis. Soc. A. ciab266. 10.1093/cid/ciab266 PMC8083549 33822014

[B93] Safaee Fakhr B. Wiegand S. B. Pinciroli R. Gianni S. Morais C. C. A. Ikeda T.. (2020). High Concentrations of Nitric Oxide Inhalation Therapy in Pregnant Patients With Severe Coronavirus Disease 2019 (COVID-19). Obstet. Gynecol. 136 (6), 1109–1113. 10.1097/AOG.0000000000004128 32852324 PMC7673637

[B94] Sasaki Y. Darmochwal-Kolarz D. Suzuki D. Sakai M. Ito M. Shima T.. (2007). Proportion of Peripheral Blood and Decidual CD4(+) CD25(bright) Regulatory T Cells in Pre-Eclampsia. Clin. Exp. Immunol. 149 (1), 139–145. 10.1111/j.1365-2249.2007.03397.x 17459078 PMC1942015

[B95] Schjetlein R. Haugen G. Wisløff F. (1997). Markers of Intravascular Coagulation and Fibrinolysis in Preeclampsia: Association With Intrauterine Growth Retardation. Acta Obstet. Gyn. Scan. 76 (6), 541–546. 10.3109/00016349709024580 9246959

[B96] Schwartz D. A. (2020). An Analysis of 38 Pregnant Women With COVID-19, Their Newborn Infants, and Maternal-Fetal Transmission of SARS-CoV-2: Maternal Coronavirus Infections and Pregnancy Outcomes. Arch. Pathol. Lab. Med. 144 (7), 799–805. 10.5858/arpa.2020-0901-SA 32180426

[B97] Shankar-Hari M. Vale C. L. Godolphin P. J.. (2021). Association Between Administration of IL-6 Antagonists and Mortality Among Patients Hospitalized for COVID-19: A Meta-Analysis. JAMA. 10.1001/jama.2021.11330 PMC8261689 34228774

[B98] Shimabukuro T. T. Kim S. Y. Myers T. R. Moro P. L. Oduyebo T. Panagiotakopoulos L.. (2021). Preliminary Findings of mRNA Covid-19 Vaccine Safety in Pregnant Persons. N. Engl. J. Med. 384 (24), 2273–2282. 10.1056/NEJMoa2104983 33882218 PMC8117969

[B99] Shin S. Jang J. Y. Roh E. Y. Yoon J. H. Kim J. S. Han K. S.. (2009). Differences in Circulating Dendritic Cell Subtypes in Pregnant Women, Cord Blood and Healthy Adult Women. J. Korean Med. Sci. 24 (5), 853–859. 10.3346/jkms.2009.24.5.853 19794983 PMC2752768

[B100] Silasi M. Cardenas I. Kwon J. Y. Racicot K. Aldo P. Mor G. (2015). Viral Infections During Pregnancy. Am. J. Reprod. Immunol. (New York N.Y. 1989) 73 (3), 199–213. 10.1111/aji.12355 PMC4610031 25582523

[B101] Straub R. H. (2007). The Complex Role of Estrogens in Inflammation. Endocr. Rev. 28 (5), 521–574. 10.1210/er.2007-0001 17640948

[B102] Szekeres-Bartho J. Faust Z. Varga P. Szereday L. Kelemen K. (1996). The Immunological Pregnancy Protective Effect of Progesterone is Manifested *via* Controlling Cytokine Production. Am. J. Reprod. Immunol. (New York N.Y. 1989) 35 (4), 348–351. 10.1111/j.1600-0897.1996.tb00492.x 8739452

[B103] Szekeres-Bartho J. Wegmann T. G. (1996). A Progesterone-Dependent Immunomodulatory Protein Alters the Th1/Th2 Balance. J. Reprod. Immunol. 31, 81–95. 10.1016/0165-0378(96)00964-3 8887124

[B104] Thevarajan I. Nguyen T. Koutsakos M. Druce J. Caly L. van de Sandt C. E.. (2020). Breadth of Concomitant Immune Responses Prior to Patient Recovery: A Case Report of non-Severe COVID-19. Nat. Med. 26 (4), 453–455. 10.1038/s41591-020-0819-2 32284614 PMC7095036

[B105] Travis M. A. Sheppard D. (2014). TGF-β Activation and Function in Immunity. Annu. Rev. Immunol. 32, 51–82. 10.1146/annurev-immunol-032713-120257 24313777 PMC4010192

[B106] Trinchieri G. (2003). Interleukin-12 and the Regulation of Innate Resistance and Adaptive Immunity. Nat. Rev. Immunol. 3 (2), 133–146. 10.1038/nri1001 12563297

[B107] Vazquez M. I. Catalan-Dibene J. Zlotnik A. (2015). B Cells Responses and Cytokine Production are Regulated by Their Immune Microenvironment. Cytokine 74 (2), 318–326. 10.1016/j.cyto.2015.02.007 25742773 PMC4475485

[B108] Veenstra Van Nieuwenhoven A. L. Heineman M. J. Faas M. M. (2003). The Immunology of Successful Pregnancy. Hum. Reprod. Update 9 (4), 347–357. 10.1093/humupd/dmg026 12926528

[B109] Virdis A. Santini F. Colucci R. Duranti E. Salvetti G. Rugani I.. (2011). Vascular Generation of Tumor Necrosis Factor-α Reduces Nitric Oxide Availability in Small Arteries From Visceral Fat of Obese Patients. J. Am. Coll. Cardiol. 58 (3), 238–247. 10.1016/j.jacc.2011.01.050 21737013

[B110] Vivier E. Raulet D. H. Moretta A. Caligiuri M. A. Zitvogel L. Lanier L. L.. (2011). Innate or Adaptive Immunity? The Example of Natural Killer Cells. Sci. (New York N.Y.) 331 (6013), 44–49. 10.1126/science.1198687 PMC3089969 21212348

[B111] Walter M. R. (2014). The Molecular Basis of IL-10 Function: From Receptor Structure to the Onset of Signaling. Curr. Top. Microbiol. 380, 191–212. 10.1007/978-3-662-43492-5_9 PMC4489423 25004819

[B112] Wang F. Hou H. Luo Y. Tang G. Wu S. Huang M.. (2020). The Laboratory Tests and Host Immunity of COVID-19 Patients With Different Severity of Illness. JCI Insight 5 (10), e137799. 10.1172/jci.insight.137799 PMC7259533 32324595

[B113] Wan Y. Shang J. Graham R. Baric R. S. Li F. (2020). Receptor Recognition by the Novel Coronavirus From Wuhan: An Analysis Based on Decade-Long Structural Studies of SARS Coronavirus. J. Virol. 94 (7), e00127–20. 10.1128/JVI.00127-20 PMC7081895 31996437

[B114] Watson J. Aarden L. A. Shaw J. Paetkau V. (1979). Molecular and Quantitative Analysis of Helper T Cell-Replacing Factors on the Induction of Antigen-Sensitive B and T Lymphocytes. J. Immunol. (Baltimore Md. 1950) 122 (5), 1633–1638. 109511

[B115] Wei L. Gao X. Chen S. Zeng W. Wu J. Lin X.. (2020). Clinical Characteristics and Outcomes of Childbearing-Age Women With COVID-19 in Wuhan: Retrospective, Single-Center Study. J. Med. Internet Res. 22 (8), e19642. 10.2196/19642 32750000 PMC7446716

[B116] Wilson E. B. Livingstone A. M. (2008). Cutting Edge: CD4+ T Cell-Derived IL-2 is Essential for Help-Dependent Primary CD8+ T Cell Responses. J. Immunol. (Baltimore Md. 1950) 181 (11), 7445–7448. 10.4049/jimmunol.181.11.7445 19017930

[B117] Wu D. Yang X. O. (2020). TH17 Responses in Cytokine Storm of COVID-19: An Emerging Target of JAK2 Inhibitor Fedratinib. J. Microbiol. Immunol. Infect. = Wei mian yu gan ran za zhi 53 (3), 368–370. 10.1016/j.jmii.2020.03.005 32205092 PMC7156211

[B118] Xiong H. Zhou C. Qi G. (2010). Proportional Changes of CD4+CD25+Foxp3+ Regulatory T Cells in Maternal Peripheral Blood During Pregnancy and Labor at Term and Preterm. Clin. Invest. Med. Medecine clinique experimentale 33 (6), E422. 10.25011/cim.v33i6.14594 21134345

[B119] Yang L. Wang J. Hui P. Yarovinsky T. O. Badeti S. Pham K.. (2021). Potential Role of IFN-α in COVID-19 Patients and its Underlying Treatment Options. Appl. Microbiol. Biot. 105 (10), 4005–4015. 10.1007/s00253-021-11319-6 PMC8096625 33950278

[B120] Yazdani Brojeni P. Matok I. Garcia Bournissen F. Koren G. (2012). A Systematic Review of the Fetal Safety of Interferon Alpha. Reprod. Toxicol. (Elmsford N.Y.) 33 (3), 265–268. 10.1016/j.reprotox.2011.11.003 22200624

[B121] Zhang Y. Liu Z. Tian M. Hu X. Wang L. Ji J.. (2018). The Altered PD-1/PD-L1 Pathway Delivers the 'One-Two Punch' Effects to Promote the Treg/Th17 Imbalance in Pre-Eclampsia. Cell Mol. Immunol. 15 (7), 710–723. 10.1038/cmi.2017.70 28890543 PMC6123412

[B122] Zhao X. Jiang Y. Zhao Y. Xi H. Liu C. Qu F.. (2020). Analysis of the Susceptibility to COVID-19 in Pregnancy and Recommendations on Potential Drug Screening. Eur. J. Clin. Microbiol. Infect. Dis. Off. Publ. Eur. Soc. Clin. Microbiol. 39 (7), 1209–1220. 10.1007/s10096-020-03897-6 PMC7178925 32328850

[B123] Zhou P. Yang X. L. Wang X. G. Hu B. Zhang L. Zhang W.. (2020). A Pneumonia Outbreak Associated With a New Coronavirus of Probable Bat Origin. Nature 579 (7798), 270–273. 10.1038/s41586-020-2012-7 32015507 PMC7095418

[B124] Zhu H. Wang L. Fang C. Peng S. Zhang L. Chang G.. (2020). Clinical Analysis of 10 Neonates Born to Mothers With 2019-Ncov Pneumonia. Trans. Pediatr. 9 (1), 51–60. 10.21037/tp.2020.02.06 PMC7036645 32154135

[B125] Ziegler C. Allon S. J. Nyquist S. K. Mbano I. M. Miao V. N. Tzouanas C. N.. (2020). SARS-CoV-2 Receptor ACE2 Is an Interferon-Stimulated Gene in Human Airway Epithelial Cells and Is Detected in Specific Cell Subsets Across Tissues. Cell 181 (5), 1016–1035. 10.1016/j.cell.2020.04.035 32413319 PMC7252096

